# Correction of GenBank’s taxonomic entry error raises a new issue regarding intergeneric relationships among salangid fishes (Osmeriformes: Salangidae)

**DOI:** 10.18699/vjgb-25-29

**Published:** 2025-04

**Authors:** E.S. Balakirev

**Affiliations:** A.V. Zhirmunsky National Scientific Center of Marine Biology, Far Eastern Branch of the Russian Academy of Sciences, Vladivostok, Russia

**Keywords:** Neosalanx, Protosalanx, taxonomic misidentification, mitochondrial genomes, CytB, single-marker sequences, genetic divergence, Neosalanx, Protosalanx, таксономические ошибки идентификации, митохондриальные геномы, CytB, маркерные последовательности, генетическая дивергенция

## Abstract

The GenBank database of publicly available nucleotide sequences is the largest genetic repository providing vitally important resources for downstream applications in biology and medicine. The concern raised about reliability of GenBank data necessitates monitoring of possible taxonomic entry errors. A case of mitochondrial genome (or mitogenome) misidentification for a salangid fish belonging to the genus Neosalanx (Osmeriformes, Salangidae) is considered in this report. The GenBank database contains four complete mitogenome sequences of N. taihuensis with the accession numbers JX524196, KP170510, MH348204, and MW291630. The overall mean p-distance for these sequences is quite high (7.01 ± 0.14 %) but becomes 29-fold lower (0.24 ± 0.05 %) after excluding the MW291630 mitogenome. An analysis of all available nucleotide sequences of salangids has shown that the observed inconsistency in the level of divergence between N. taihuensis mitogenomes is due to species misidentification. It has turned out that the mitogenome MW291630 available in GenBank does not belong to N. taihuensis, but is, in fact, a mitogenome of N. jordani misidentified as N. taihuensis. The resolved taxonomic identity of the MW291630 mitogenome, as well as an extended sample of species with investigated single-marker sequences, has raised some new issues regarding intergeneric relationships in salangid fishes. In particular, the obtained data do not support synonymization of the genus Neosalanx with Protosalanx, as was suggested in the last revision of the salangid classification. As the comparative analysis of interspecific and intergeneric divergences shows, Protosalanx is not an all-inclusive clade that includes all Neosalanx species. Instead, it consists of (at least) two evolutionary distinct lineages with the level of genetic divergence between them matching well the mean value of divergence between the other salangid genera. Further analysis using nuclear genome-wide data is required to have new insights into the evolution of salangid fishes.

## Abstract (ru)

Генетическая база данных GenBank является крупнейшим генетическим хранилищем, предо- ставляющим жизненно важные ресурсы для последующего применения в биологии и медицине. Выска- занная обеспокоенность надежностью GenBank обусловливает необходимость мониторинга возможных таксономических ошибок в записях этой базы данных. Здесь мы сообщаем о случае ошибочной идентифи- кации митохондриального генома (или митогенома) у саланксовой рыбы, принадлежащей к роду Neosalanx (Osmeriformes, Salangidae). База данных GenBank содержит четыре полные последовательности митогенома N. taihuensis с номерами доступа JX524196, KP170510, MH348204 и MW291630. Средняя p-дистанция между этими последовательностями довольно велика (7.01 ± 0.14 %), но становится в 29 раз меньше (0.24 ± 0.05 %) после исключения митогенома MW291630. Анализ всех доступных нуклеотидных последовательностей са- лангид показал, что наблюдаемое несоответствие в уровне дивергенции между митогеномами N. taihuensis обусловлено ошибочной идентификацией видов. Оказалось, что митогеном MW291630 не принадлежит N. taihuensis, а в действительности представляет митогеном N. jordani, ошибочно идентифицированный как N. taihuensis. Установленная таксономическая идентичность митогенома MW291630, а также расширенная выборка видов с исследованными маркерными последовательностями выявили некоторые новые аспекты межродовых отношений у саланксовых рыб. В частности, полученные данные не подтверждают синоними- зацию рода Neosalanx с Protosalanx, как это было предложено в последней ревизии классификации салан- гид. Как показывает настоящий анализ, Protosalanx не является кладой, включающей все виды Neosalanx. Напротив, эта клада состоит по крайней мере из двух эволюционно разных линий, уровень генетической дивергенции между которыми соответствует межродовым значениям дивергенции у салангид. Необходим дальнейший анализ с использованием полных ядерных геномов для выяснения эволюции саланксовых рыб.

## Introduction

The value and reliability of the GenBank database (Sayers et al., 2023) depends on the accuracy of species identification of biological samples, which is quite often not provided when based solely on morphology with an insufficient number of diagnostic characters. Species identification errors have been increasingly referred to as a serious challenge limiting the utility and reliability of public databases. In fact, for organisms such as fungi, which are notoriously difficult to distinguish, up to 20 % (Nilsson et al., 2006) or even 30 % (Hofstetter et al., 2019) of DNA sequence records in GenBank may have erroneous lineage designations. Multiple taxonomic misidentifications were reported for nuclear genome-sequenced strains of medically important lower eukaryotes (e. g., Houbraken et al., 2021), for single-marker sequences of many fishes (e. g., Li et al., 2018), and for complete mitogenomes of many higher eukaryotes, including bivalve mollusks (Salvi et al., 2021; Cunha et al., 2022), ticks (Mohamed et al., 2022), insects (Ožana et al., 2022; Kim et al., 2023), parasitic nematodes (Nielsen et al., 2014), fishes (Cheng et al., 2012; Balakirev et al., 2017, 2024; Oleinik et al., 2019; Sangster, Luksenburg, 2021a; Teske, 2021), amphibians (Mulder et al., 2016), reptiles (Simonov et al., 2018), birds (Sangster, Luksenburg, 2021b), and placental mammals (Botero-Castro et al., 2016).

A taxonomic misidentification causes discordance between the species name and the nucleotide sequence, thus, compromising downstream inferences. Consequently, it is urgently important to disclose such problematic sequences and report them as fast as possible after their deposition in GenBank in order to prevent propagation of incorrect biological information among databases and subsequent publications (e. g., Balakirev et al., 2017, 2024; Sangster, Luksenburg, 2021b).

Here, we report a case of mitochondrial genome misidentification for a salangid fish belonging to the genus Neosalanx Wakiya, Takahashi, 1937 (Osmeriformes, Salangidae). Salangids are endemic to East Asia and inhabit a wide range of marine, brackish-water, and freshwater habitats in China, Vietnam, Korean Peninsula, Japan, and Russia (e. g., Roberts, 1984). These are small, neotenic fishes with early maturation, relatively high fecundity, and a life span of about one year. Species identification of salangid fishes remains a serious challenge.

The taxonomy of salangids, based on morphological, ecological, and genetic approaches, has been subject to various revisions with multiple known synonyms (Fu et al., 2005, 2012; Zhang et al., 2007; Guo et al., 2011). In particular, it was shown that N. taihuensis Chen, 1956, N. tangkahkeii (Wu, 1931), and N. pseudotaihuensis Zhang, 1987 are junior synonyms of N. brevirostris (Pellegrin, 1923) (Zhang et al., 2007; Guo et al., 2011). Hemisalanx Regan, 1908 was shown to be a junior synonym of Salanx Cuvier, 1816 (Guo et al., 2011). Somewhat close genetic relationships were also found (Zhang et al., 2007) between Protosalanx chinensis (Basilewsky, 1855), N. anderssoni (Rendahl, 1923), N. argentea (Lin, 1932), and N. tangkahkeii. Based on the morphological characters, ecological preferences, and genetic data (mitochondrial CytB gene), Zhang et al. (2007) identified a group of species within the genus Neosalanx, including N. reganius Wakiya, Takahashi, 1937, N. jordani Wakiya, Takahashi, 1937, N. oligodontis Chen, 1956, and Neosalanx sp., which they proposed to treat as a separate new undescribed genus “Microsalanx”. Zhang et al. (2007) and Guo et al. (2011) assumed that N. anderssoni may also belong to the genus Protosalanx Regan, 1908. Using extensive morphological analysis and also genetic markers such as mitochondrial (CytB) and seven nuclear genes, Fu et al. (2012) suggested that the genus Neosalanx should be considered a junior synonym of Protosalanx. These authors also found a distant relationship between Salangichthys ishikawae Wakiya, Takahashi, 1913 and S. microdon Bleeker, 1860, which proved that the two species belong to different genera: Salangichthys Bleeker, 1859 and the newly established Neosalangichthys Fu, Li, Xia, Lei, 2012 including a single species, N. ishikawae. Fu et al. (2012) found that the genera Leucosoma Gray, 1831 and Salanx differ significantly in genetic and morphological diagnostic characters and are, therefore, not synonymous.

Yang et al. (2020) deposited a complete mitogenome of the salangid N. taihuensis to GenBank under the accession no. MW291630 (taxonomy ID NCBI:txid240825), providing the forth mitogenome for this species in addition to the already available ones: JX524196, KP170510, and MH348204. An analysis of the new N. taihuensis MW291630 mitogenome in comparison with all other available mitogenome sequences, as well as the use of single-marker sequences of salangid fishes, has shown that this mitogenome sequence does not belong to N. taihuensis. We found that the specimen investigated by Yang et al. (2020) was erroneously identified as N. taihuensis and actually represents N. jordani. Therefore, the aim of the present study was to document this GenBank entry error and use the correctly identified MW291630 mitogenome, as well as an extended sample of single-marker sequences, to clarify some challenging issues regarding intergeneric relationships among salangid fishes

## Materials and methods

Mitochondrial genomes and single-marker sequences

A total of 13 complete mitogenome sequences from fishes of the family Salangidae Bleeker, 1859 were accessed from the Genetic Sequence Data Bank (the National Center for Biotechnology Information; NCBI-GenBank Flat File Release 260.0, April 15, 2024). The outgroup species, including Plecoglossus altivelis (Temminck, Schlegel, 1846) (family Plecoglossidae Bleeker, 1859) and Retropinna retropinna (Richardson, 1848) (family Retropinnidae Gill, 1862), were selected based on the previous molecular evidence of their close relationship to the family Salangidae (Fu et al., 2005; Zhang et al., 2007; Guo et al., 2011) and on a screening of nucleotide sequences available in GenBank using the basic local alignment search tool (BLAST) procedure (Altschul et al., 1990). Additionally, we also analyzed 406 mitochondrial single-marker sequences, including 12S rRNA, 16S rRNA, ND1, COI, and CytB published in previous studies on salangids (see Supplementary Table S1 for accession numbers and references)1.


Supplementary Materials are available in the online version of the paper: https://doi.org/10.5281/zenodo.13455533


DNA sequence analysis. Previously, we described the DNA sequence analysis in detail elsewhere (e. g., Balakirev et al., 2017, 2020; Balakirev, 2022). The main steps are summarized in brief below. The nucleotide sequences were aligned using the software MUSCLE (Edgar, 2004). The programs DnaSP v. 6 (Rozas et al., 2017) and MEGA v. 11 (Tamura et al., 2021) were used for intra- and interspecific analysis of polymorphism and divergence based on uncorrected p-distance (Kartavtsev, 2011; Collins et al., 2012). Phylogenetic reconstructions were inferred from an analysis of complete mitogenomes by the maximum likelihood methods available in IQ-TREE v. 2 (Nguyen et al., 2015). The trees were constructed using complete mitogenomes or mitochondrial single-marker sequences only (12S rRNA, 16S rRNA, ND1, COI, and CytB). For all reconstructions, the best-fit model of nucleotide substitution was chosen with the Akaike Information Criterion and the Bayesian Information Criterion in MEGA and IQ-TREE. The ultrafast maximum likelihood bootstrap analysis (Hoang et al., 2018) consisted of 10,000 replicates.

## Results and discussion

Variability and divergence of salangid mitogenomes

Figure 1 displays a maximum likelihood tree of complete mitogenome sequences for the salangid species including representatives of the valid genera Salanx, Leucosoma, Salangichthys, Protosalanx, and Neosalanx. The tree shows the N. taihuensis (with synonyms) specimens present in two significantly diverged clusters (Lineage 1 and Lineage 2; Fig. 1) with the overall mean distance equal to 7.01 ± 0.14 %. The N. taihuensis mitogenome sequences from Lineage 1 (JX524196, KP170510, and MH348204) were very similar to each other (with an average p-distance of 0.24 ± 0.05 %), thus, demonstrating a typical level of intraspecific nucleotide diversity in fishes (e. g., Kartavtsev et al., 2016; Li et al., 2018). Lineage 1, except for N. taihuensis, also included P. chinensis and N. anderssoni. The overall mean distance between the species from Lineage 1 (using a single randomly picked sequence per species) was 7.70 ± 0.17 % with pairwise p-distances varying from 4.82 ± 0.17 % between P. chinensis and N. anderssoni to 9.21 ± 0.22 % between P. chinensis and N. taihuensis, which matched well the known interspecific nucleotide diversity in fishes (e. g., Kartavtsev et al., 2016; Li et al., 2018). Lineage 2 (Fig. 1) included the N. taihuensis MW291630 mitogenome only, which demonstrated a high level of divergence (14.08 ± 0.27 %) with the representative sequence of the N. taihuensis mitogenome from Lineage 1. With the use of all mitogenomes for the species from Lineage 1 for comparison (P. chinensis, N. anderssoni, and N. taihuensis; Fig. 1), the difference between Lineages 1 and 2 still remained markedly higher (13.78 ± 0.24 %) than the overall mean distance (7.70 ± 0.17 %) estimated for Lineage 1

**Fig. 1.**
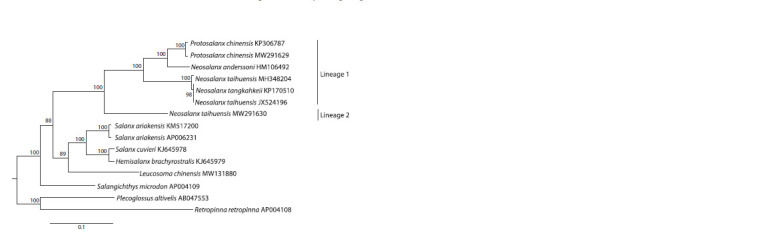
Maximum likelihood tree inferred from an analysis of the complete mitochondrial genomes for fishes of the family Salangidae The TIM2+F+I+G4 model was used to construct the tree. The numerals at the nodes are bootstrap probability (percentage) values based on 10,000 replicates (values lower than 75 % are omitted). The tree includes all salangid mitogenomes available in GenBank except the three recombinant sequences of Protosalanx chinensis under the accession nos. HM106494, MH330683, and KJ499917 (Balakirev, 2022). The synonymous species names N. taihuensis or N. tangkahkeii were used for the originally published KP170510, MW291630, JX524196, and MH348204 mitogenomes. To avoid any confusion, we leave the names as they were originally assigned for the salangid species considered in this paper.

We found the diagnostic 15-bp deletion that occurs within the ND5 gene (at coordinates 79–93, Supplementary Fig. S2) and the 1-bp and 2-bp diagnostic deletions that occur within the non-coding (control) region (at coordinates 534, 963, 1051–1052, and 1071; Supplementary Fig. S3). These are shared by the P. chinensis, N. anderssoni, and N. taihuensis mitogenomes (Lineage 1) and distinguish them clearly from the N. taihuensis MW291630 (Lineage 2) and the rest of the salangid mitogenomes. The 15-bp deletion within the ND5 gene is the only sequence length variability detected for the protein-coding genes in the mitogenomes of salangid fishes. Taking into account the high phylogenetic informativeness of gaps (e. g., Giribet, Wheeler, 1999), these diagnostic deletions provide robust evidence for the close relationships of the species belonging to Lineage 1 and their distinct difference from Lineage 2.

To scale the value of full mitogenome divergence between Lineages 1 and 2, we estimated the average level of divergence based on the representative genera including Protosalanx, Salanx, Leucosoma, and Salangichthys. To be conservative, we excluded N. anderssoni and N. taihuensis (with synonyms) in order to prevent underestimation of p-distance values due to possible congeneric relationships of these species (Zhang et al., 2007; Guo et al., 2011). We also excluded the MW291630 mitogenome sequence with uncertain identity. The obtained overall mean p-distance for all available genera of salangid fishes was 14.87 ± 0.21 % with pairwise p-distances varying from 12.51 ± 0.26 % between Leucosoma and Salanx to 17.04 ± 0.29 % between Leucosoma and Protosalanx (Table 1), which was close to the value of divergence between Lineages 1 and 2 (13.78 ± 0.24 %).

**Table 1.**
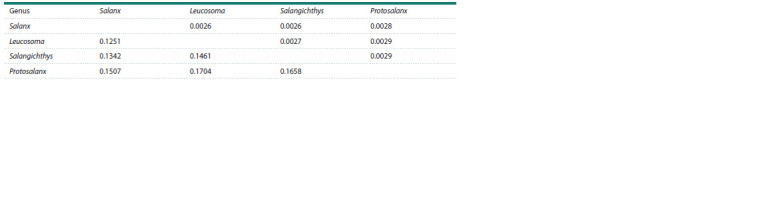
Pairwise p-distances between salangid genera based on complete mitogenomes Note. The salangid genera Salanx, Leucosoma, Salangichthys, and Protosalanx are represented by the following species: Salanx ariakensis Kishinouye, 1902 (AP006231), Leucosoma chinensis (Osbeck, 1765) (MW131880), Salangichthys microdon (AP004109), and Protosalanx chinensis (KP306787). The p-distances are below the diagonal line. The standard errors, obtained with 10,000 bootstrap replications, are above the diagonal line.

It is worth noting that in pairwise comparisons the divergence between Lineages 1 and 2 (13.78 ± 0.24 %) was not markedly different from the divergence between Leucosoma and Salangichthys, or was even slightly higher than the divergence between Leucosoma and Salanx, as well as between Salangichthys and Salanx (Table 1). Thus, the pairwise comparisons showed that the mitogenome divergence between Lineage 1 and Lineage 2 matched well the intergeneric level of divergence in salangid fishes. The interlineage distance matched also the average value of divergence between different genera reported for the single-marker sequences or complete mitogenomes in other groups of fishes (e. g., Kartavtsev et al., 2016; Li et al., 2018; Balakirev et al., 2020).

Identification of the MW291630 mitogenome

According to a taxonomic hypothesis based on genetic data, N. taihuensis, N. tangkahkeii, N. pseudotaihuensis, and N. brevirostris are synonyms (Zhang et al., 2007; Guo et al., 2011). Consequently, the genus Neosalanx is represented in GenBank by only two species, N. taihuensis (with synonyms) and N. anderssoni (HM106492; Supplementary Table S1), which makes the identification of the problematic complete mitogenome MW291630 impossible. However, the GenBank database contains at least five more Neosalanx species, representing the full taxonomic diversity known for the genus Neosalanx, that were investigated using mitochondrial singlemarker sequences: N. argentea, N. jordani, N. oligodontis, N. reganius, and Neosalanx sp. (the names of the species are listed as they were identified by the authors who submitted the respective nucleotide sequences to GenBank). The nucleotide sequences obtained for these species can be used to resolve the observed inconsistency detected for the N. taihuensis complete mitogenomes and to identify the taxonomically problematic MW291630 mitogenome

We analyzed the GenBank mitochondrial single-marker sequences that are most frequently used in taxonomic and phylogenetic reconstructions of salangid fishes, including 12S rRNA, 16S rRNA, ND1, COI, and CytB. A preliminary analysis revealed that among the single-marker sequences, only CytB demonstrated noticeable divergence values. The other markers provided much lower resolution but were still not contradictory to the CytB data (see, e. g., the maximum likelihood tree based on the COI gene; Supplementary Fig. S1). Consequently, further analysis was based on the CytB gene only.

Figure 2 illustrates the maximum likelihood tree based on the CytB gene for N. taihuensis and other members of the family Salangidae representing almost the entire taxonomic diversity of the genus Neosalanx. There were two significantly different clusters that included the species name N. taihuensis. These clusters corresponded to Lineages 1 and 2 identified on the basis of mitogenome sequences (Fig. 1). The overall mean p-distance for Lineage 1 was 9.13 ± 0.66 % using a single randomly picked sequence per species (with pairwise p-distances varying from 3.33 ± 0.54 % between N. taihuensis and N. argentea to 12.09 ± 0.99 % between N. taihuensis and N. anderssoni). Lineage 1 included P. chinensis and part of the Neosalanx species (N. anderssoni, N. taihuensis, and N. argentea) that Fu et al. (2012) had combined with other Neosalanx species and synonymized with the genus Protosalanx (see Introduction). The second cluster contained N. oligodontis, N. reganius, N. jordani, Neosalanx sp., and the CytB portion of the MW291630 mitogenome with an overall mean p-distance of 4.69 ± 0.45 % and pairwise p- distances varying from 2.10 ± 0.43 % between N. oligodontis and N. reganius to 7.01 ± 0.76 % between N. reganius and Neosalanx sp. This cluster included a group of species that were placed in the genus “Microsalanx” erected by Zhang et al. (2007). The mean p- distance between the clusters (Lineage 1 and Lineage 2, Fig. 2) was 19.03 ± 1.06 % with a single randomly picked sequence per species or 18.97 ± 1.09 % with all 146 sequences available for Lineages 1 and 2 (Supplementary Table S1). This value fit well into the range of intergeneric divergences of fishes (e. g., Kartavtsev et al., 2016; Li et al., 2018; Balakirev et al., 2020).

**Fig. 2.**
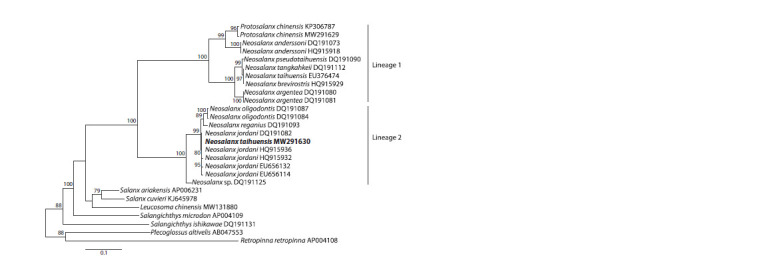
Maximum likelihood tree for the members of the family Salangidae based on the CytB gene sequences The Tamura-Nei + gamma (TN93+G) model was used to infer the tree. The N. jordani CytB sequences are represented by three datasets investigated by Fu et al. (2012) (HQ915932 and HQ915936), Zhang et al. (2007) (DQ191082), and Zhao et al. (2010) (EU656114 and EU656132). The N. taihuensis MW291630 mitogenome is highlighted in bold. For tree reconstruction, we used only some representative samples from larger datasets (a full list of the CytB sequences is provided in Supplementary Table S1). For other comments, see Figure 1.

An analysis of pairwise p-distances showed a surprisingly high level of sequence divergence (18.89 ± 1.16 %) (Table 2) between the GenBank CytB sequences of N. taihuensis, including 70 isolates obtained from different localities by various authors (Zhang et al., 2007; Zhao et al., 2008; see also Supplementary Table S1) with low intraspecific variability (0.44 ± 0.09 %), and the respective CytB portion of the MW291630 mitogenome. The result was consistent with the data obtained for the complete mitogenomes (see above), which showed a substantial difference between the MW291630 mitogenome and the other N. taihuensis (with synonyms) mitogenomes, JX524196, KP170510, and MH348204, available in GenBank.

**Table 2.**
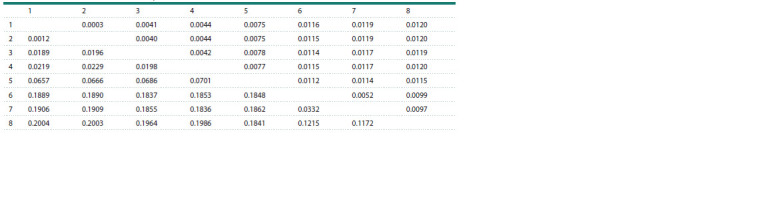
Pairwise p-distances between the CytB sequences for Neosalanx species Note. All available CytB sequences for each included species were used for this analysis (the number of sequences is in parentheses). 1: MW291630 (1); 2: N. jordani (25); 3: N. oligodontis (7); 4: N. reganius (1); 5: Neosalanx sp. (1); 6: N. taihuensis (70); 7: N. argentea (2); and 8: N. anderssoni (10). For other comments, see Table 1 and Figure 2.

The CytB portion of the N. taihuensis MW291630 mitogenome demonstrated very close affinity to the N. jordani singlemarker sequences obtained from different localities by various authors (25 isolates; Zhang et al., 2007; Zhao et al., 2010; Fu et al., 2012) with low intraspecific variability (0.24 ± 0.05 %; see also Fig. 2). The p-distance between the CytB gene of the MW291630 mitogenome and the GenBank CytB sequences of N. jordani was surprisingly low (0.12 ± 0.03 %; Table 2); it fit well into the range of intraspecific variability in fishes (e. g., Kartavtsev et al., 2016; Li et al., 2018). The maximum likelihood tree based on the COI gene showed a similar topology (Supplementary Fig. S1).

Three species, N. oligodontis, N. reganius, and Neosalanx sp., demonstrated more pronounced differences from the MW291630 mitogenome than N. jordani (Table 2, Fig. 2). Zhang et al. (2007) suggested that N. reganius and N. oligodontis could be considered as subspecies of N. jordani. Indeed, N. jordani, N. oligodontis, N. reganius, Neosalanx sp., and the CytB portion of the MW291630 mitogenome were all in the same cluster (Fig. 2) with an overall mean p-distance of 4.69 ± 0.45 %, which suggested close relationships for these salangid species.

Thus, the single-marker sequences clearly showed that the GenBank accession no. MW291630 represents the mitogenome of N. jordani mistaken for N. taihuensis. The observed inconsistency in the level of divergence between the N. taihuensis mitogenomes (see above) is due to incorrect species identification. The CytB analysis of within- and between lineage variability confirmed the data obtained with complete mitogenomes.

MtDNA data indicates a generic level of divergence between Lineage 1 and Lineage 2

The close relationships between N. jordani, N. oligodontis, N. reganius, and Neosalanx sp. had been reported previously, as well as the significant difference of this group from other Neosalanx and Protosalanx species including N. taihuensis, N. argentea, N. anderssoni, and P. chinensis (Fig. 2, Table 2) (Zhang et al., 2007; Guo et al., 2011). Based on integrative data, Zhang et al. (2007) erected the genus “Microsalanx” (see Introduction). The results of the present analysis do not contradict this hypothesis. Both the complete mitogenomes (Fig. 1) and the single-marker sequences (Fig. 2) clearly demonstrated two significantly diverged clusters (Lineage 1 and Lineage 2). The interlineage divergence based on the CytB gene was high (18.97 ± 1.09 %). It was significantly higher than the average divergences within each of the lineages: the overall mean distances for Lineage 1 and Lineage 2 separately were 9.13 ± 0.66 and 4.69 ± 0.45 %, respectively.

To evaluate the scale of divergence between Lineages 1 and 2, we estimated the average level of divergence based on the CytB gene for the salangid genera available in GenBank including Protosalanx, Salanx, Leucosoma, Neosalangichthys, and Salangichthys (Table 3). The obtained overall mean p- distance was 16.98 ± 0.78 % (with pairwise p-distances varying from 11.74 ± 0.93 % between Leucosoma and Salanx to 21.47 ± 1.25 % between and Protosalanx and Salangichthys; Table 3), which was close to the value of divergence between Lineages 1 and 2 (18.97 ± 1.09 %) based on the multiple CytB gene sequences (see above). The divergence between Lineages 1 and 2 (18.97 ± 1.09 %) was not markedly different from that between Protosalanx and Salanx or it was even higher than the p-distances in pairwise comparisons of Leucosoma vs. Salanx, Neosalangihthys, and Salangichthys; Salanx vs. Neosalangihthys and Salangichthys; and Neosalangihthys vs. Salangichthys (Table 3).

**Table 3.**
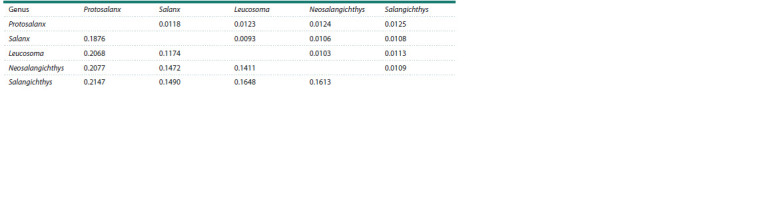
Pairwise p-distances between salangid genera based on the CytB gene Note. The salangid genera Protosalanx, Salanx, Leucosoma, Neosalangichthys, and Salangichthys are represented by the following species: Protosalanx chinensis (KP306787), Salanx ariakensis (AP006231), Leucosoma chinensis (MW131880), Neosalangichthys ishikawae (Wakiya, Takahashi, 1913) (DQ191127), and Salangichthys microdon (AP004109). For other comments, see Table 1.

Thus, an analysis of the multiple CytB sequences encompassing the full diversity of salangid fishes showed a high level of divergence between Lineage 1 and Lineage 2 (18.97 ± 1.09 %), which fit into the range of intergeneric distances reported for salangids (see above) and other fishes (see the references above). Lineage 1 included a group of species (P. chinensis, N. anderssoni, N. taihuensis, and N. argentea; Fig. 2) comprising a part of the reorganized genus Protosalanx (Fu et al., 2012). The group of species from Lineage 1 was previously divided in two sub-groups (“primitive lineages”) (Zhang et al., 2007; Guo et al., 2011). Indeed, the pairwise p-distances for Lineage 1 varied within a relatively wide range from 3.32 ± 0.52 % between N. taihuensis and N. argentea to 12.15 ± 0.99 % between N. taihuensis and N. anderssoni (Table 2). However, the overall mean sub-group divergence (P. chinensis + N. anderssoni vs. N. taihuensis + N. argentea) within Lineage 1 was still markedly lower (11.42 ± 0.87 %) than the divergence between Lineages 1 and 2 (18.97 ± 1.09 %). Thus, unlike Zhang et al. (2007) and Guo et al. (2011), we did not find sufficient grounds to split Lineage 1 into two sub-groups and consider it a single evolutionary lineage representing the genus Protosalanx. This conclusion was supported by the diagnostic deletions detected within the ND5 gene and the control region in the salangids’ mitogenomes (see the “Variability and divergence of salangid mitogenomes” section above). Nevertheless, the relationships between the “primitive lineages” P. chinensis + N. anderssoni and N. taihuensis + N. argentea need to be further clarified using a more representative array of genetic markers (see below).

Lineage 2 contained a group of species (N. oligodontis, N. reganius, N. jordani, and Neosalanx sp.) placed in the genus “Microsalanx” by Zhang et al. (2007). This subdivision was reasonable (see Introduction) to distinguish this group of species from the rest of the Neosalanx species. However, the transfer of N. taihuensis (with synonyms), N. anderssoni, and N. argentea to the genus Protosalanx, as suggested earlier (Zhang et al., 2007; Guo et al., 2011; Fu et al., 2012) and supported by our data (Figs. 1 and 2, Tables 1 and 2), gives reason to abolish the genus name “Microsalanx” (at least until the generic heterogeneity is proven for Lineage 1; see above). Consequently, the original genus name Neosalanx is appropriate for the salangid species N. oligodontis, N. reganius, N. jordani, and Neosalanx sp. comprising Lineage 2 (Figs. 1 and 2).

Thus, in contrast to Fu et al. (2012), our analysis based on complete mitogenomes and mtDNA single-marker sequences, as well as the analysis of Zhang et al. (2007) based on morphological, ecological, and genetic data, did not support the synonymization of all Neosalanx species with Protosalanx. The data clearly show two substantially diverged evolutionary lineages (Figs. 1 and 2): (1) P. chinensis, N. anderssoni, N. taihuensis (with synonyms), and N. argentea representing the genus Protosalanx and (2) N. oligodontis, N. reganius, N. jordani, and Neosalanx sp. representing the genus Neosalanx.

For phylogenetic analysis of salangid fishes, Fu et al. (2012) used a concatenated multigene dataset including the mitochondrial CytB gene and seven nuclear sequences (28S rRNA, RAG1, zic1, ENC1, RNF213, glyt, and SH3PX3). As a result (among others), these authors (Fu et al., 2012, p. 853) discovered that “all species from the ‘Neosalanx–Protosalanx’ complex belong to a same genus” and considered Neosalanx as a junior synonym of Protosalanx.

Compared to mtDNA markers and complete mitogenomes, the nuclear markers (28S rRNA, RAG1, zic1, ENC1, RNF213, glyt, and SH3PX3), mostly used by Fu et al. (2012), demonstrated a much lower divergence between the salangid genera. For the genera Protosalanx (except Neosalanx), Salanx, Leucosoma, Neosalangichthys, and Salangichthys, the values of the overall mean distance for the nuclear markers were low and varied in a narrow range (from 1.98 ± 0.36 % for zic1 to 3.56 ± 0.54 % for RAG1). The low divergence of the nuclear markers can be explained by the fact that they mostly represent highly conserved sequences developed for analyzing deep phylogenetic relationships on a scale of dozens to hundreds of millions of years, e. g., to infer phylogenetic relationships of all bony fishes, which requires analysis of genomic regions with slow rates of evolution (e. g., Betancur-R et al., 2017). These markers might be not sensitive enough for salangid fishes that experienced most speciation events around 1.1–9.9 Ma (Zhang et al., 2007). As a consequence, we suggest that the phylogenetic signal of CytB, also used by Fu et al. (2012), was significantly “diluted” by the effect of strongly conserved nuclear sequences. Indeed, the overall mean p-distance between the genera Protosalanx (except Neosalanx), Salanx, Leucosoma, Neosalangichthys, and Salangichthys was equal to 16.98 ± 0.78 % based on the CytB gene only (see above). However, it decreased significantly, to 2.72 ± 0.17 %, when the nuclear multigene dataset of Fu et al. (2012) was used.

Although the suggested relationships in salangid fishes seem robust, we expect them to be modified, possibly, as new genetic data become available. In particular, the mitochondrial sequences have revealed a relatively high level of divergence between two sub-groups within the genus Protosalanx (P. chinensis + N. anderssoni and N. taihuensis + N. argentea; Fig. 2) (see also Zhang et al., 2007; Guo et al., 2011), which may indicate a supra-species taxonomical range. Consequently, more nuclear genome-wide data are necessary to further address this and other issues concerning the taxonomic composition and the evolutionary relationships among salangid fishes.

## Conclusion

Misidentified nucleotide sequences, including complete mitogenomes, are becoming increasingly frequent in GenBank, which leads to an explosive spread of incorrect biological information in subsequent scientific publications over time

The misidentified N. taihuensis MW291630 mitogenome has been revealed in our study. We argue that the GenBank accession no. MW291630 actually represents the mitogenome of N. jordani mistaken for N. taihuensis. Thus, GenBank users should be aware of the above-described entry error to avoid conflicting results in their downstream evolutionary and comparative genomic studies

The data obtained have raised a new issue regarding intergeneric relationships among salangid fishes. In contrast to the study by Fu et al. (2012), our data from the comparative analyses of interspecific and intergeneric divergences do not support the synonymization of the genus Neosalanx with Protosalanx and oppose the suggestion to consider Neosalanx as a junior synonym of Protosalanx. Genome-wide studies are needed to further clarify the evolutionary relationships of salangid fishes.

The introduction and spread of misidentified nucleotide sequences in genetic databases, which compromises downstream applications, is unlikely to be completely curbed. However, some appropriate steps can be undertaken (see, e. g., Balakirev et al., 2017, 2024; Sangster, Luksenburg, 2021b) to minimize their massive accumulation and subsequent propagation in scientific publications, thereby increasing the reliability of findings reported in them.

## Conflict of interest

The authors declare no conflict of interest.
